# Evaluation of various sample sources for the cytologic diagnosis of *Cytauxzoon felis*

**DOI:** 10.1111/jvim.16338

**Published:** 2021-12-02

**Authors:** Casey R. Sleznikow, Jennifer L. Granick, Leah A. Cohn, Laura A. Nafe, Aaron Rendahl, Erin N. Burton

**Affiliations:** ^1^ University of Minnesota Veterinary Medical Center St. Paul Minnesota USA; ^2^ Department of Veterinary Clinical Sciences University of Minnesota St. Paul Minnesota USA; ^3^ Department of Veterinary Medicine and Surgery University of Missouri Columbia Missouri USA; ^4^ Department of Biochemical and Veterinary Sciences University of Minnesota St. Paul Minnesota USA

**Keywords:** cytauxzoonosis, piroplasm, schizont

## Abstract

**Background:**

*Cytauxzoon felis* is a life‐threatening protozoan disease of cats. Identification of schizont‐laden macrophages is a point‐of‐care diagnostic test for acute cytauxzoonosis.

**Hypothesis/Objectives:**

The primary objective determined cytologic agreement between sample types to diagnose acute cytauxzoonosis. The secondary objective evaluated novices' ability to identify cytauxzoon organisms in blood films and tissue aspirates.

**Animals:**

Thirty‐eight cats with suspected acute cytauxzoonosis and 5 controls examined postmortem.

**Methods:**

Cases were prospectively submitted and collected. Blood film, lymph node, and splenic aspirates were blindly reviewed for sample quality, presence of schizont‐laden macrophages, and agreement between sample types. A subset of cases and controls were evaluated by 12 blinded novice observers to determine sensitivity and specificity for identifying organisms in various sample types.

**Results:**

Acute cytauxzoonosis diagnosis was made on at least 1 sample type in 28/38 cats. Schizont‐laden macrophages were seen on 33% (10/30) of blood films, 56% (19/34) lymph node aspirates, 77% (26/34) splenic aspirates. Schizont‐laden macrophages were more likely seen on splenic than lymph node aspirates (McNemar's, *P* = .03) or blood film (McNemar's, *P* = <.001). Novice observers were more likely to agree with experts when identifying schizont‐laden macrophages in splenic aspirates (sensitivity = 77.1%, specificity = 94.4%) versus lymph node aspirates (sensitivity = 52.8%, specificity = 96.4%) or blood films (sensitivity = 41.7%, specificity = 96.9%).

**Conclusions and Clinical Importance:**

Schizont‐laden macrophages are most frequently identified in spleen, even by novice observers. If the diagnosis of acute cytauxzoonosis cannot be confirmed via blood film, then splenic, followed by peripheral lymph node aspirates can be considered.

**Abbreviations**
BCP: board certified clinical pathologist
hpf: high powered field
IMR: internal medicine resident
lpf: low powered field

## 1 INTRODUCTION

Cytauxzoonosis is an acute, life‐threatening illness in domestic cats caused by the apicomplexan protozoan parasite *Cytauxzoon felis* that is transmitted by the *Amblyomma americanum* tick.^1^ Wild cats, mainly the bobcat (*Lynx rufus*), are the natural reservoirs and domestic cats that survive infection could become persistently infected.^2^, ^3^, ^4^, ^5^

Acute cytauxzoonosis occurs when schizont‐laden macrophages become distended and lodge in small blood vessels, resulting in multiorgan failure and often death of the infected cat. Clinical signs of schizogony include high fever, lethargy, inappetence, icterus, peripheral lymphadenomegaly, and hepatosplenomegaly. As the disease progresses, schizont‐laden macrophages rupture and release merozoites that are taken up by red blood cells through endocytosis. Although anemia often accompanies initial erythrocyte infection, cats that survive the schizogenous stage of infection often maintain low levels of intraerythrocytic merozoites (piroplasms) indefinitely without apparent illness and become persistently infected.^5^, ^6^

A commercial PCR test, sensitive and specific for disease detection preceding cytologically detectable parasitemia, is available. Previous PCR tests detecting 18S ribosomal RNA did not differentiate between cats with acute cytauxzoonosis and the chronic carrier state.^5^, ^7^, ^8^ Newer methods target mitochondrial genes which are present in higher copy numbers than 18S during acute infection.^9^ These newer methods could allow differentiation between acute cytauxzoonosis and the chronic carrier state. However, the 1 to 2 day turnaround for PCR testing often negates the usefulness for initial diagnosis.^10^

**FIGURE 1:**
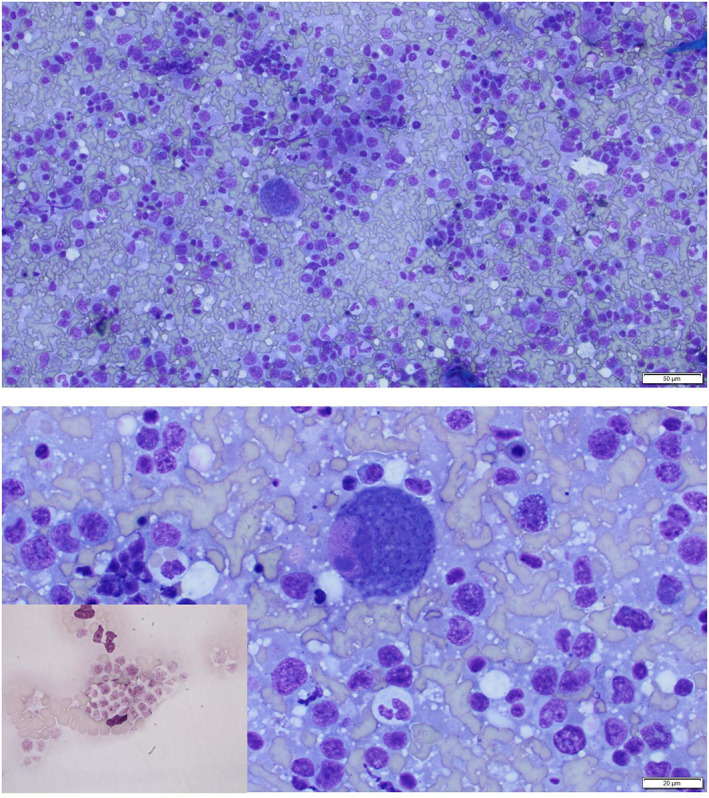
Images of splenic aspirates with schizont‐laden macrophages at 20× objective (top) and 50× objective (bottom; Modified Wright Giemsa). An example of clumped feline platelets at 100× objective are seen on the inset in the bottom image for comparison. The scale bar is not applicable to the inset

Delay in diagnosis and treatment of cytauxzoonosis can prove fatal.^11^ The only point‐of‐care test for acute cytauxzoonosis is microscopic identification of schizont‐laden macrophages in blood or tissue aspirates. Piroplasms are seen in both acute and chronic infections, are small (approximately 1‐2 μm) and can be mistaken for Howell Jolly bodies, stain precipitate, or other organisms. Additionally, during the acute phase of disease piroplasms might be present in low numbers.^1^ In contrast, schizonts are the predominant life stage present during acute cytauxzoonosis, and schizont‐laden macrophages can be observed in aspirates of various tissues (spleen, lymph node, liver) as well as the feathered edge of blood films.^12^ Schizont‐laden macrophages are large (typically ranging from 30 to 50 μm) and have a distinct appearance, making them less likely to be mistaken for other cell types or artifacts (Figure 1), although it is possible that inexperienced observers could mistake them for platelet clumps if not examined with high magnification.

Unlike the limitations of both the PCR test and the identification of piroplasms, identification of the schizont‐laden macrophages would provide a practitioner an immediate diagnosis of acute cytauxzoonosis allowing them to feel more confident when talking with the owner regarding the potential overall cost of therapy and prognosis. The primary objective of this study was to determine the agreement between the evaluation of blood films, splenic aspirate, and lymph node aspirate samples to support a diagnosis of acute cytauxzoonosis. The secondary objective was to evaluate a novice's ability, when compared to clinicians with more advanced training in clinical pathology, to identify piroplasms or schizont‐laden macrophages in blood films and tissue aspirates collected from domestic cats with a high clinical suspicion for acute cytauxzoonosis.

## 2 MATERIALS AND METHODS

General practitioners in Oklahoma, Missouri, Tennessee, and Arkansas who previously participated in *C. felis* research were contacted to participate in this study. Clinicians were asked to collect 3 sample types from cats suspected to have acute cytauxzoonosis and that died or were euthanized on the day of presentation. Samples requested included 2 blood films, aspirates from any lymph node (preferably peripheral node for ease of sampling), and splenic aspirates. Written informed client consent was granted before collection of tissue and blood samples. As disseminated intravascular coagulation (DIC) is common in cats with cytauxzoonosis, we requested only postmortem samples to avoid iatrogenic bleeding from tissue aspirates.^13^ This study was exempt from the University of Minnesota's Institutional Animal Care and Use Committee approval, as all samples were collected as part of normal clinical work‐up or postmortem. Peripheral blood samples for blood film preparations were requested to be collected immediately postmortem or from lab work performed the day of presentation. Samples were shipped to the investigators who stained all slides with a modified Wright‐Giemsa stain (Siemens Hema‐tek 2000; Wescor Aerospray 7120).

In order to address the primary objective—to determine the agreement between blood film evaluation, splenic aspirate, and lymph node aspirate for diagnosis of acute cytauxzoonosis—all slides were evaluated by 2 observers with more advanced training in clinical pathology and interobserver agreement was calculated (see statistics section below). The 2 observers were a board certified clinical pathologist (BCP) and a third‐year internal medicine resident (IMR). There was no time limit for scanning the slides for any observer. Observers were blinded to each other's results. Blood films were evaluated for all stages of disease (ie, piroplasms and schizont‐laden macrophages). The monolayer was defined as an area of the blood films in which the red blood cells were evenly distributed with infrequent touching and no large spaces without cells. The monolayer was evaluated at high power using the 100× objective lens for the presence of piroplasms. If piroplasms were visualized, an average number per high powered field (hpf) was determined by counting the number of organisms/hpf for 10 fields. Blood films were evaluated for schizont‐laden macrophages at low power using the 10× objective lens. The entire slide was evaluated for schizont‐laden macrophages. Slides prepared from the splenic and lymph node aspirates were evaluated for cellularity and the severity of peripheral blood contamination; a scoring system (Table S1) was adapted from a previously described splenic aspirate scoring system.^14^ The entire slides of splenic and lymph node preparations were evaluated for the presence or absence of schizont‐laden macrophages.

After completion of individual review of samples by BCP and IMR for the purpose of determining interobserver agreement, samples for which disagreement was present were reviewed by both observers simultaneously to reach a consensus. If a consensus could not be reached a third expert, also a boarded clinical pathologist, evaluated the slide independently. Interobserver agreement was determined by comparison of identification of all stages of disease; however, the diagnosis of acute cytauxzoonosis was based on the presence of schizont‐laden macrophages in any tissue type. Expert observer consensus was then used to make comparisons between sample types for the detection of schizont‐laden macrophages.

To address the secondary aim of this study—to evaluate a novice's ability to identify schizont‐laden macrophages as well as piroplasms—12 additional novice observers, all veterinary professional students, reviewed 10 complete slide sets for the presence or absence of organisms. Five cases were selected randomly from all study cases, regardless of presence or absence of schizont‐laden macrophages, and 5 cases were selected from historical submissions to the University of Minnesota's clinical pathology service to serve as negative controls. Negative control cytology and blood films came from cats residing outside of endemic regions that had no travel history (presumably *C. felis* negative). The 10 cases were then organized using a random number generator for each novice. Novices were given a brief training on general cytology techniques and organism identification by BCP. Along with the training, the novices were provided with positive control cytology samples from each sample site and textbook resources for reference. No time limit for evaluation of the samples was implemented for the novices. Novice agreements (to identify piroplasms and schizont‐laden macrophage in blood film and schizont‐laden macrophages only in tissue aspirates), sensitivity, and specificity in comparison to the consensus data of BCP and IMR were determined.

### 2.1 Statistics

To assess agreement of the 2 experienced readers, both overall agreement and Cohen's kappa were computed. A *P* < .05 was considered statistically significant. Kendall's rank correlation coefficient was used to determine the association between the degree of cellularity and blood contamination and the presence of organisms on the slide. To address the first objective, comparing the presence of organisms in blood films, and splenic and lymph node aspirates, pairwise McNemar's tests were performed, using consensus data of BCP and IMR to assess if organisms were more likely to be seen in blood films, lymph node or splenic aspirates. To address the second objective, the sensitivity and specificity of the novices' ability to identify organisms were calculated for each sample type (blood film, lymph node aspirate, and splenic aspirate) and the expert consensus data was used as the gold standard for comparison. The 95% confidence intervals were calculated for each of those proportions using a bootstrap method. R version 3.6.1 was used for all computations.

## 3 RESULTS

### 3.1 Study sample and collection type

Diagnostic samples were submitted from 38 cats ranging in age from 8 weeks to 15 years. Though cat signalment data was not available for all submissions, both male (5 neutered and 2 intact) and female (1 spayed and 3 intact) cats were included. Submissions originated from Missouri (n = 15), Tennessee (5), Oklahoma (6), Arkansas (3), or unknown (9). The 3 requested sample types (blood films, lymph node, and spleen) were not available for all submissions. Complete sample sets (blood films, lymph node, and spleen) were available for 26 cases. A total of 99 total cytology sites (blood films, lymph node, and spleen) submitted for evaluation (Table S2).

### 3.2 Expert agreement of the detection of *C. felis*

When all available slides were evaluated independently by both of the experienced observers (BCP and IMR), the overall agreement on the presence or absence of *C. felis* for all sample sites (blood film, lymph node, and spleen) evaluated was 94.9% (kappa = .88). IMR and BCP disagreed on 5/98 samples, including 2 splenic aspirates, 2 lymph node aspirates, and 1 blood film. There was disagreement regarding the presence of a single organism on 1 blood film; IMR observed the single piroplasm that BCP did not. A second expert (BCP), who was not blinded to the previous results, was consulted to reach a consensus and a single piroplasm was identified on the entire blood film. For the remainder of samples in which there was disagreement BCP saw the organisms and IMR did not. When those samples were reviewed together a consensus was reached that the organism was truly present. When individual sample types were compared, the overall agreement between IMR and BCP for the blood film, lymph node aspirates, and splenic aspirates was 96.7%, 94.1%, and 94.1%, respectively (Table 1).

**TABLE 1:** Agreement between BCP and IMR for various sample sources

	BCP		IMR			
	Present	Absent	Present	Absent	Interobserver agreement	Kappa
Blood (n = 30)	23	7	24	6	96.7%	.9
Lymph node (n = 34)	19	15	17	17	94.1%	.88
Spleen (n = 34)	26	8	26	8	94.1%	.84

Abbreviations: BCP, board certified clinical pathologist; IMR, internal medicine resident.

### 3.3 Impact of sample type and cellularity on detection of schizont‐laden macrophages

Using the consensus data, a Kendall's rank correlation coefficient was used to determine if the degree of cellularity and blood contamination correlated with the presence of organism on the slide. Only the degree of cellularity of the splenic samples was statistically significant (rho = .34, *P* = .04); increased nucleated cells on a slide increased the likelihood of observing schizont‐laden macrophages in splenic aspirates. In other words, the more cellular the aspirate the more likely schizont‐laden macrophages were observed.

### 3.4 Detection of acute cytauxzoonosis in blood films and FNA samples

The diagnosis of acute cytauxzoonosis was made on at least 1 sample type in 28/38 cats. Schizont‐laden macrophages were seen on 10 out of the available 30 submitted blood films (33%), 19/34 (56%) lymph node aspirates and 26/34 (77%) splenic aspirates. On all blood films in which schizont‐laden macrophages were observed, piroplasms were also observed. For 8 of these blood films in which the diagnosis of acute cytauxzoonosis could be made, both lymph node and splenic aspirates were also available; schizont‐laden macrophages were present on both lymph node and splenic aspirates of 6 cases. Of the 2 remaining cases schizont‐laden macrophages were present on the splenic aspirates but not the lymph node aspirates.

Of the 20 blood films in which the diagnosis of acute cytauxzoonosis was not able to be made, 18 had both lymph node and splenic aspirates available for review. In 14 of these 18 the diagnosis of acute cytauxzoonosis could be made. Schizont‐laden macrophages were present in both lymph node and splenic aspirates in 11/18 cases and present in splenic aspirates only in 3/18. The remaining 4/18 cases with complete slide sets were negative for schizonts‐laden macrophages in all samples. Of the 2 cases in which there were not complete data sets, a lymph node aspirate (n = 1) was submitted and was negative for shizont‐laden macrophages, or neither were submitted (n = 1).

### 3.5 Comparison of sample types for the detection of acute cytauxzoonosis

When lymph node samples were compared to blood films for the presence of schizont‐laden macrophages, there were 6 cats in which schizont‐laden macrophages were present in both blood and lymph node, and there were 8 cats in which they were absent in both sample sources. There were 11 cats in which schizont‐laden macrophages were present in the lymph node and absent in the blood film and 3 cats in which they were absent in the lymph node but present on the blood film. Therefore schizont‐laden macrophages were not more likely to be found in lymph node aspirates compared to blood films (McNemar's, *P* = .06) (Table 2).

**TABLE 2:** McNemar's test results

Lymph node				Lymph node				Spleen			
		Yes	No			Yes	No			Yes	No
Blood	Yes	6	3	Spleen	Yes	19	6	Blood	Yes	8	0
	No	11	8		No	0	7		No	14	4
	*P* = .06				*P* = .03				*P* = <.001		

*Note*: For blood smear, lymph node and spleen, “yes” indicates the identification of schizont‐laden macrophages, “no” is the absence of schizont‐laden macrophages.

When lymph nodes and splenic aspirates were compared, 6 cats had schizont‐laden macrophages that were observed in the splenic aspirate but not the lymph node. There were no instances in which there were schizont‐laden macrophages detected in the lymph node and not in the spleen. Schizont‐laden macrophages were more likely to be found in splenic aspirates than lymph node aspirates (McNemar's, *P* = .03).

Finally, when splenic aspirates and blood films were compared for schizont‐laden macrophages there were 4 cats in which schizont‐laden macrophages were absent in both blood and spleen and there were 8 cats in which they were present in both sample sources. There were 14 cases in which the schizont‐laden macrophages were present in the spleen and absent in the blood film and there were no cats in which schizont‐laden macrophages were present on the blood film and absent in splenic aspirates. Schizont‐laden macrophages were more likely to be found in splenic aspirates than on blood film review (McNemar's, *P* < .001).

### 3.6 Novice ability to identify acute cytauxzoonosis

The novices' ability to identify organisms in various sample sources compared to the expert observers, the overall agreement, sensitivity, and specificity for each sample source and the calculated 95% confidence intervals are summarized in Table 3. The novice's overall agreement with the experts did not vary widely between sample sources.

**TABLE 3:** Overall agreement between novice observers and experts and sensitivity and specificity of novice observers for various sample sources

	Blood (piroplasms)	Blood (schizonts)	Lymph node (schizonts)	Spleen (schizonts)
Specificity (95% CI)	88.9% (75.0‐98.6)	96.9% (89.6‐100)	96.4% (88.9‐100)	94.4% (79.2‐100)
Sensitivity (95% CI)	81.2% (50.0‐100%)	41.7% (0‐83.3)	52.8% (3.5‐91.7)	77.1% (52.8‐95.8)
Overall agreement (95% CI)	85.8% (70.8‐95.8)	85.8% (65.8‐99.2)	83.3% (63.3‐98.3)	87.5% (74.2‐97.5)

Abbreviation: CI = confidence interval.

The sensitivity and specificity of the novice's ability to identify organisms did vary widely for each sample source. Two of the 5 cases that the novices evaluated that were randomly selected from the complete datasets had schizont‐laden macrophages, in addition to piroplasms on the blood films. Overall the novices' sensitivity for detecting piroplasms in the blood (81.2%) was higher than their sensitivity to detect schizont‐laden macrophages in the blood (41.7%). The sensitivity of the novice observers identifying the presence of schizont‐laden macrophages in lymph node aspirates was 52.8% and 77.1% in splenic aspirates. Overall, the novices' were more likely to identify schizont‐laden macrophages in the spleen compared to any other sample type.

## 4 DISCUSSION

Acute cytauxzoonosis is the result of the schizogenous replication of the parasite in mononuclear cells and therefore illness can occur before piroplasms are identifiable within the red blood cells.^15^, ^16^ On the day of presentation the sensitivity of cytologic identification of piroplasms for diagnosis of infection can be as low as 50%.^1^, ^17^ Because schizogenous replication occurs during the earliest stages of illness, it has been speculated that aspirates of lymph nodes or spleen could be more sensitive for the early diagnosis of infection.^18^ Identification of schizont‐laden macrophages in tissue aspirates or blood film is a feasible point‐of‐care test for the antemortem diagnosis of acute cytauxzoonosis.

The first objective of this study was to determine the agreement between various sample sources; blood film, splenic and lymph node aspirates for the diagnosis of acute cytauxzoonosis. The lack of confirmatory molecular testing and inclusion of cats without cytologic evidence of *C. felis* organisms in any sample precludes us from determining sensitivity and specificity of sample types for diagnosis of acute cytauxzoonosis. Schizont‐laden macrophages were seen on 33 (10/30) of all available blood films. The identification of these schizont‐laden macrophages on the blood film allows for a rapid diagnosis of acute cytauxzoonosis and allows the practitioner to forgo potentially more invasive diagnostics such as splenic or lymph node aspirates. When individual sample sources were compared using McNemar's test, schizont‐laden macrophages were more likely to be found in splenic aspirates than lymph node aspirates. In cases in which schizont‐laden macrophages cannot be identified using blood film alone, based on these results, splenic aspirates followed by peripheral lymph node aspirates could be used by practitioners to identify the schizont‐laden macrophages.

Antemortem aspiration of the spleen is still an uncommon procedure in most primary care clinics but is becoming a more common practice as the cost of ultrasound units continues to decrease, and teleradiology and mobile diagnostic imaging services become more widely available in both urban and rural settings. This change will likely result in an increased comfort level and frequency of acquiring splenic aspirates in cats with suspected acute cytauxzoonosis. It should be noted that acquisition of splenic aspirates is not without risk. Disseminated intravascular coagulation is common in cats with naturally occurring cytauxzoonosis.^13^ Additionally, cats with concurrent confirmed severe thrombocytopenia could also be at increased risk for hemorrhage. However, in 1 small study of 5 cats with cytauxzoonosis, spontaneous or excessive bleeding was not reported in any cat before or during hospitalization, or following invasive procedures, despite having clinicopathologic data supportive of DIC.^13^ Given the potential clotting abnormalities, the decision to perform a splenic aspirate must be made on a case‐by‐case basis along with informed client consent. If splenic aspirates are deemed too risky for the cat, aspiration of a peripheral lymph node could be considered with the understanding that schizont‐laden macrophages can be seen in peripheral lymph node samples, but not as frequently as the spleen.

The secondary aim of the study was to evaluate a novice's ability to identify piroplasms or schizont‐laden macrophages in blood films and tissue aspirates from cats with high index of suspicion for acute cytauxzoonosis. The novice observers identified the organisms with relatively high frequency and relatively high agreement with the experts. The specificity of the novice's ability to identify schizont‐laden macrophages on blood film and in lymph nodes and splenic aspirates was slightly higher than their ability to identify piroplasms on blood films. This was not a surprising finding, as piroplasms are significantly smaller (1‐2 μm) in size than the intact schizont‐laden macrophages (typically ranging from 30 to 50 μm) observed in the splenic and lymph node aspirates. Piroplasms can be easily mistaken for stain precipitant, Howell‐Jolly bodies, artifacts, or potentially overlooked with low parasitemia. The sensitivity of the novices' identification of schizont‐laden macrophages was highest in splenic aspirates compared to lymph node and blood films. When schizont‐laden macrophages were identified on blood film, they were typically found along the feathered edge and were sometimes broken, making them more challenging to diagnose. Additional training on identification of schizont‐laden macrophages in blood films and the knowledge that they can be identified in some cases of acute cytauxzoonosis could be helpful to diagnose these cases with the least invasive means necessary.

There are several limitations of this small study. First, an important limitation to this study is that definitive diagnosis of *C. felis* by a combination of PCR, cytology, and postmortem examination was not performed. Samples were collected based upon a strong clinical suspicion for acute cytauxzoonosis by general practitioners in endemic regions. However, cytologic evaluation failed to identify *C. felis* organisms in one quarter (10/38) of the cats in this study, and so it is possible that these cats were suffering from diseases other than acute cytauxzoonosis. The lack of molecular diagnostic and postmortem confirmation of infection in all patients precluded our ability to determine the sensitivity and specificity of the various sample sources to rapidly diagnose acute cytauxzoonosis. Future studies evaluating cytologic diagnosis of acute cytauxzoonosis would benefit from concurrent PCR testing of collected blood or tissues, as well as postmortem histologic evaluation of formalin‐fixed tissues, including spleen, lymph node, liver, and lung.

A second limitation of the study is that although acute cytauxzoonosis is a systemic disease affecting multiple organs, clinicians were not asked to collect samples from visceral organs aside from the spleen, nor were they asked to collect samples from a consistent anatomic lymph node (eg, popliteal lymph node). In this study, 12 of the 38 cats lacked sampling of all 3 evaluated tissues. For future studies and in clinical work up of cats suspected to have acute cytauxzoonosis, we recommend sampling and evaluation of blood, spleen, and lymph node, as well as liver, if possible. This approach would allow for the most thorough cytologic assessment. Future studies could also investigate whether the anatomical location of the lymph node(s) sampled or the degree of lymphadenomegaly impacts the likelihood of seeing schizont‐laden macrophages in aspirates.

A third limitation was that the sample size was relatively low and might have been inadequate to identify differences in the presence of schizont‐laden macrophages between blood smears and lymph node aspirates. Additionally, the novice observers only evaluated a small number of cases. Further studies are needed in which novice observers evaluate a larger number of cases to determine sensitivity and specificity for identification of schizont‐laden macrophages in various sample sources.

In conclusion, if schizont‐laden macrophages are not identified in blood films, splenic then lymph node aspiration should be considered as the next point‐of‐care diagnostic step for the diagnosis of acute cytauxzoonosis. Novice observers were able to identify schizont‐laden macrophages most readily in splenic aspirates, followed by lymph node aspirates and blood films.

## CONFLICT OF INTEREST DECLARATION

Authors declare no conflict of interest.

## OFF‐LABEL ANTIMICROBIAL DECLARATION

Authors declare no off‐label use of antimicrobials.

## INSTITUTIONAL ANIMAL CARE AND USE COMMITTEE (IACUC) OR OTHER APPROVAL DECLARATION

Informed consent was obtained from the owner or legal custodian of all animals included of all animal(s) described in this work for the procedure(s) undertaken.

## HUMAN ETHICS APPROVAL DECLARATION

Authors declare human ethics approval was not needed for this study.

## Supporting information


**Table S1** Scoring system for cellularity and blood contamination of splenic and lymph node aspirates.

**Table S2** Summary of the BCP and IMR consensus data on the presence or absence of organisms in various sample types.
