# Supplementary material for: Evaluation of various sample sources for the cytologic diagnosis of Cytauxzoon felis
Source: J Vet Intern Med. 2021 Dec 2;36(1):126–32. doi: 10.1111/jvim.16338 (PMC8783339; doi:10.1111/jvim.16338)
Supplement: Supplementary file 1 — Table S1 Scoring system for cellularity and blood contamination of splenic and lymph node aspirates. [file JVIM-36-126-s002.pdf]

Supplementary Table 1: Scoring system for cellularity and blood contamination of splenic and lymph node aspirates.

**Cellularity**	3	High number of nucleated cells
	2	A moderate number of nucleated cells, in addition to the number expected with the degree of blood contamination
	1	Very few nucleated cells, in addition to the number expected with the degree of blood contamination
	0	Acellular
**Blood contamination**	3	Significant blood contamination that impaired the ability to evaluate the nucleated cells present
	2	Moderate amount of blood contamination but did not impact the ability to evaluate the nucleated cells present
	1	Rare red blood cells
	0	None
