# Supplementary material for: Evaluation of various sample sources for the cytologic diagnosis of Cytauxzoon felis
Source: J Vet Intern Med. 2021 Dec 2;36(1):126–32. doi: 10.1111/jvim.16338 (PMC8783339; doi:10.1111/jvim.16338)
Supplement: Supplementary file 2 — Table S2 Summary of the BCP and IMR consensus data on the presence or absence of organisms in various sample types. [file JVIM-36-126-s001.pdf]

Supplementary Table 2. Summary of the BCP and IMR consensus data on the presence or absence of organisms in various sample types.

Case number	Blood (schizont)	Blood (piroplasm)	Spleen (schizont)	Lymph node (schizont)
1	N	N	N	N
2	N	Y	Y	Y
3	Y	Y	Y	Y
4	Y	Y	Y	N
5	NA	NA	N	NA
6	NA	NA	N	N
7	NA	NA	Y	Y
8	Y	Y	NA	NA
9	N	Y	Y	N
10	Y	Y	Y	Y
11	NA	NA	Y	Y
12	Y	Y	Y	Y
13	N	Y	Y	Y
14	N	N	N	N
15	N	N	N	N
16	N	N	NA	NA
17	Y	Y	Y	N
18	Y	Y	Y	Y
19	N	Y	Y	Y
20	N	N	NA	N
21	N	Y	Y	Y
22	N	N	N	N
23	N	Y	Y	N
24	Y	Y	NA	N
25	Y	Y	Y	Y
26	N	Y	Y	Y
27	N	Y	Y	N
28	NA	NA	N	N
29	NA	NA	N	N
30	NA	NA	Y	NA
31	NA	NA	Y	N
32	Y	Y	Y	Y
33	N	Y	Y	Y

34	N	Y	Y	Y
35	N	Y	Y	Y
36	N	Y	Y	Y
37	N	Y	Y	Y
38	N	Y	Y	Y

Y= organism present, N= organism absent, NA= sample not available

BCP= board certified clinical pathologist, IMR= internal medicine resident
